# Olfactory bulb plasticity ensures proper olfaction after severe impairment in postnatal neurogenesis

**DOI:** 10.1038/s41598-017-05970-1

**Published:** 2017-07-18

**Authors:** D. Díaz, R. Muñoz-Castañeda, C. Ávila-Zarza, J. Carretero, J. R. Alonso, E. Weruaga

**Affiliations:** 1 Laboratory of Neuronal Plasticity and Neurorepair, Institute for Neuroscience of Castile & Leon (INCyL), Universidad de Salamanca, Salamanca, Spain; 2 Institute of Biomedical Research of Salamanca, IBSAL, Salamanca, Spain; 3 Applied Statistics Group, Department of Statistics, Universidad de Salamanca, Salamanca, Spain; 4 Department of Human Anatomy and Histology, Faculty of Medicine, University of Salamanca, Salamanca, Spain; 5 Instituto de Alta Investigación, Universidad de Tarapacá, Arica, Chile

## Abstract

The olfactory bulb (OB) neurons establish a complex network that ensures the correct processing of the olfactory inputs. Moreover, the OB presents a lifelong addition of new neurons into its existing circuitry. This neurogenesis is considered essential for the OB function. However, its functional impact on physiology and behavior is still unclear. Here, we investigate the mechanisms of OB plasticity that underlie bulbar physiology in relation to severe damage of neurogenesis. The neurogenesis of young mice was altered by ionizing radiation. Afterwards, both multi-channel olfactometry and electrophysiological studies were performed. Furthermore, neurogenesis and differentiation of the newly formed cells were assessed using bromodeoxyuridine labeling combined with a wide battery of neuronal markers. Our results demonstrate a reduction in both neurogenesis and volume of the OB in irradiated animals. The number of neuroblasts reaching the OB was reduced and their differentiation rate into interneurons selectively changed; some populations were noticeably affected whereas others remained preserved. Surprisingly, both olfactory detection and discrimination as well as electrophysiology presented almost no alterations in irradiated mice. Our findings suggest that after damaging postnatal neurogenesis, the neurochemical fate of some interneurons changes within a new biological scenario, while maintaining homeostasis and olfaction.

## Introduction

The olfactory bulb (OB) is the first cerebral relay structure in the olfactory pathway where olfaction is processed^1, 2^. In rodents, it is formed by different layers where neurons receive, modify and send the olfactory information to higher olfactory cortices^3^. The olfactory inputs are generated by the olfactory sensory neurons of the olfactory epithelium, whose axons reach the OB. Once there, these axons synapse in the olfactory glomeruli with the apical dendrites of both mitral and tufted cells, which transmit the sensory information outside of the OB^4^. The activity of these projecting neurons is refined and modulated by different groups of interneurons, principally by periglomerular and granule cells^1, 2^. Moreover, the OB is a neurogenic structure: neuroblasts coming from the subventricular zone (SVZ) after travelling throughout the rostral migratory stream (RMS) reach the OB to become new interneurons^1, 5–8^. This continuous delivery is necessary to replace the old interneurons, establishing a continuous cell turnover that seems to be essential for the correct functioning of the OB, and consequently for olfaction^6, 9–11^.

This cell turnover has a remarkable complexity and the neuronal fate of neuroblasts is determined within the SVZ^12^, giving rise to a wide range of different populations of interneurons^13^. The function of some of these populations has been previously analyzed^14–17^, but the functioning of this complex neuronal network is not fully understood. Moreover, cell turnover depends on the developmental stage of the animal, thus defining at least two periods of neurogenesis: postnatal (up to around 30 days of life, P30) and adult (from P30 onwards)^18^. Although cell turnover is present in both periods and changes are gradual, cell proliferation and integration are higher in young animals as compared to adults^18–20^. Furthermore, the OB can modify the various parameters of cell turnover depending on environmental cues, sensorial conditions, molecular and hormonal signals or after an injury^21–23^, which adds to its remarkable plasticity.

Ionizing radiation is amongst the most striking factors that can affect adult neurogenesis, since proliferating cells are highly susceptible to this type of damage^24^. Different studies have addressed the effects of ionizing radiation on neurogenic regions, including the SVZ, the RMS and the OB, which oscillate between a lack of recovery and the complete restoration of neurogenesis (for a review see ref. 25). Intriguingly, after certain doses -high enough to deplete cell proliferation without subsequent recovery- a remnant of neurogenesis in the SVZ-RMS-OB still persists throughout time, even when it is postnatally disrupted^26^. In addition, after such doses of radiation the olfactory ability of animals is not affected^27–30^, something which is remarkable considering: 1) the arrival of new neurons is severely reduced^26, 29^; 2) the OB shows a dramatic shrinkage^26, 29^; and 3) these postnatally-generated neurons are considered essential for OB function^6, 9–11^. Consequently, some mechanisms of compensatory plasticity may occur after severe injury to ensure proper functioning of the OB, especially if the damage takes place during postnatal development.

The purpose of the present study was to analyze changes in plasticity in the OB after a significant reduction in postnatal neurogenesis. More precisely, we sought to determine whether changes in the residual cell turnover -after severe ionizing radiation- could be the basis for maintaining general bulbar homeostasis.

## Results

### Radiation severely affects OB volume

**Figure 1:**
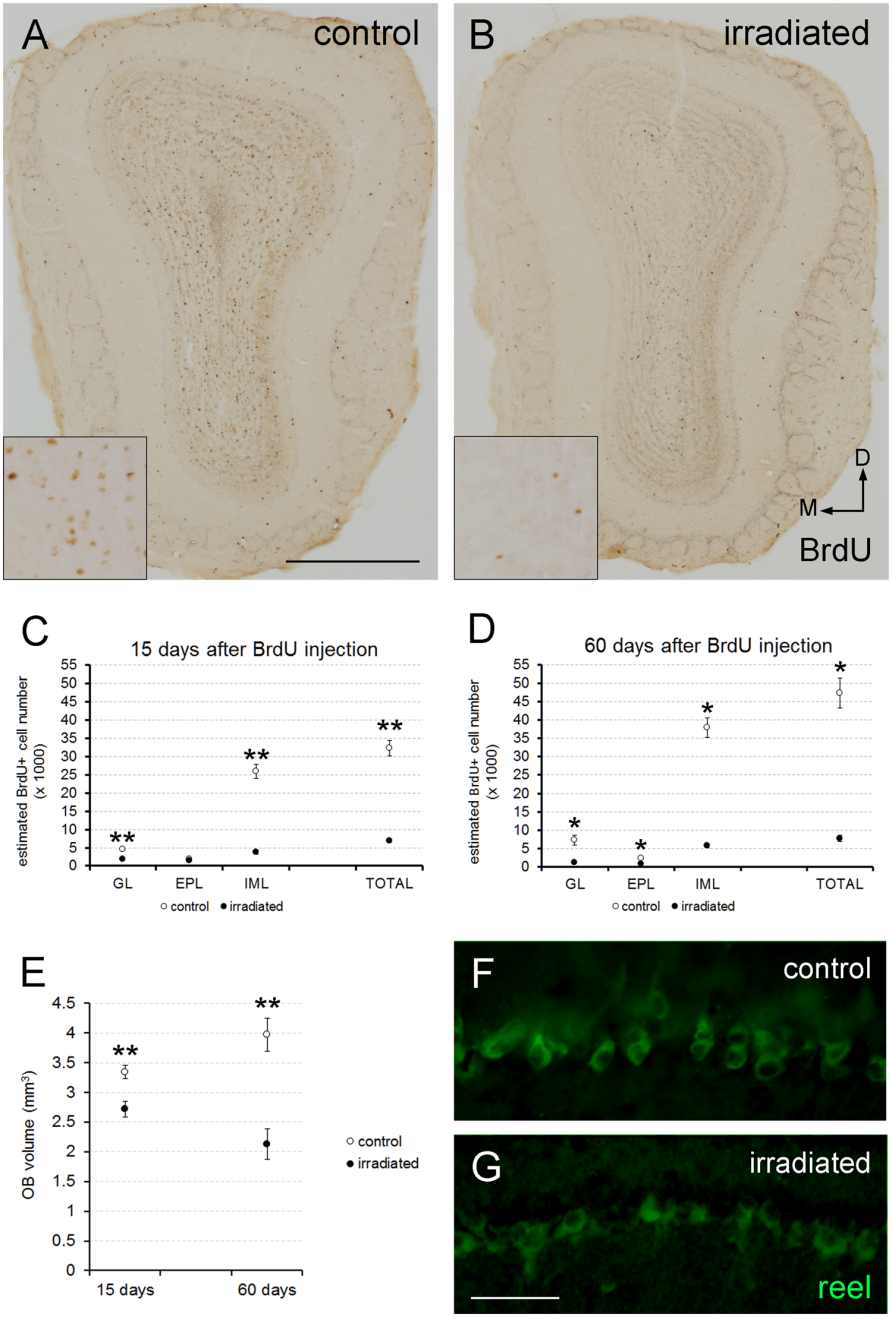
Effect of radiation in cell turnover, OB volume and mitral cells. (**A**,**B**) Coronal slices of the OB from control and irradiated mice 15 days after BrdU injection; note the reduction in BrdU staining; inset panels enlarge representative areas to show the differences between both situations. (**C**,**D**) Estimation of BrdU-positive cells in the different OB layers 15 (**C**) and 60 days (**D**) after BrdU injection; note the reduction in the number of cells in the irradiated animals except for the EPL 15 days after BrdU injection. (**E**) Estimation of the OB volume; note the dramatic reduction in irradiated animals at both survival times. (**F**,**G**) Mitral cells of both experimental groups stained with reelin (green); no differences were detected. M, medial; D, dorsal. *p < 0.05; **p < 0.01. Scale bar 500 μm for (**A**,**B**); 100 μm for (**F**,**G**).

Our first analysis was aimed at estimating the volume of the analyzed portion of the OB (containing all the bulbar layers and without including both the accessory olfactory bulb and the anterior olfactory nucleus; see supplementary material), which appeared reduced in irradiated mice at both survival ages (15 days, p = 0.008; 60 days, p = 0.008; Fig. 1E, Supp. Fig. 3), that is 30 and 75 days after radiation (i.e., 15 and 60 days after injections of  bromodeoxyuridine, BrdU). The volume reduction was also present in the bulbar layering (for 15 days: glomerular layer, GL, p = 0.109; external plexiform layer, EPL, p = 0.006; inframitral layers, IML, p = 0.003; for 60 days: GL, p = 0.021; EPL, p = 0.007; IML, p = 0.001; Supp. Fig. 3). These findings demonstrated that radiation triggers an effect in the size of the OB more dramatically and sooner than expected (see Discussion below). However, these volumetric changes in size did not affect the method for counting cells, as the volume was estimated within each of the OB layers analyzed.

### Radiation severely affects OB cell turnover

Following on, we decided to estimate the number of BrdU-positive cells that reached the different layers of the OB after lethal radiation. Fifteen days after BrdU injection, the total number of BrdU-positive cells was markedly lower in irradiated animals (p = 0.008; Fig. 1A–C). This difference was evident in both GL (p = 0.008) and IML (p = 0.008; Fig. 1C), while the EPL was unaffected (p = 0.222; Fig. 1C). Sixty days after BrdU injection, the differences in the number of newly generated cells between control and irradiated animals persisted in all of the layers of the OB analyzed, also including the EPL (p = 0.036 for all comparisons; Fig. 1D).

Furthermore, from 15 to 60 days after BrdU injection, the estimated number of BrdU-immunostained cells in control mice increased in both GL (p = 0.036) and IML (p = 0.036; Supp. Fig. 4A,C) but not in EPL (p = 0.571; Supp. Fig. 4B). Conversely, in irradiated mice both GL and IML remained unchanged (GL, p = 0.056; IML, p = 0.095; Supp. Fig. 4A,C), whereas the EPL showed a decrease in BrdU-positive cells (p = 0.016; Supp. Fig. 4B). Finally, the estimated total number of BrdU-labeled cells increased between both survival times (15 and 60 days) in control animals (p = 0.036; Supp. Fig. 4D) whereas it remained unchanged in irradiated mice (p = 0.548; Supp. Fig. 4D). In any case, 60 days after BrdU injection neuroblasts reached the OB of the irradiated animals (Fig. 1D), and a residual cell turnover persisted.

Additionally, we analyzed the population of the principal type of projecting neurons: the mitral cells. Immunohistochemical analysis showed that radiation did not seem to affect the mitral cell number (Fig. 1F,G), a result which fits with what was observed in the electrophysiological analyses (see below). Moreover, and as we might have expected, no BrdU labeling was detected in any of the mitral cells of any experimental group at any survival time, since these are prenatally generated^1, 31^.

### Radiation differentially affects subtypes of new interneurons

Since newly born cells still reached the OB after severe radiation, the next step was to determine possible changes in their subtypes. Thus, we combined BrdU labeling with a battery of specific neuronal markers for several neuronal populations of the OB.

**Figure 2:**
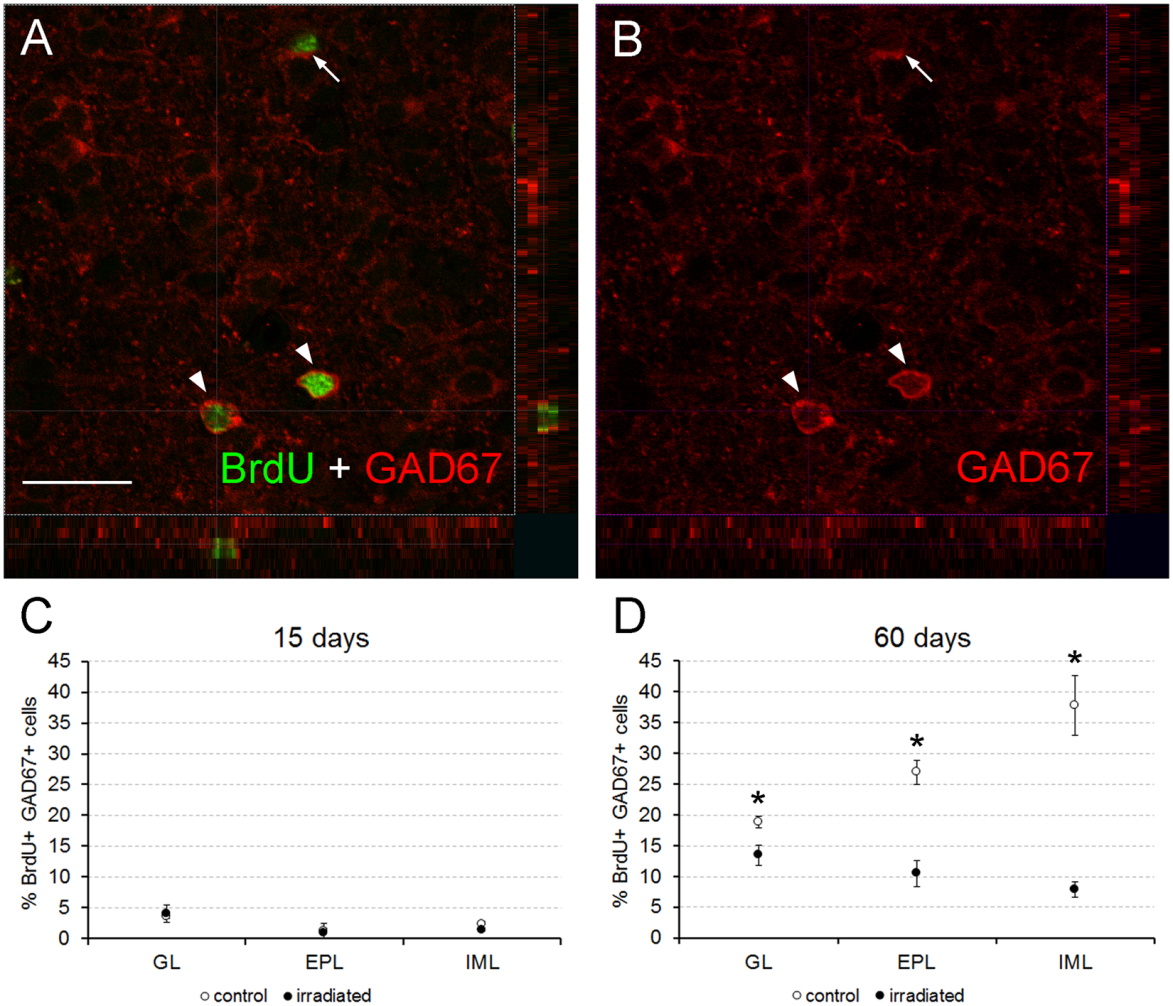
Cell characterization. (**A**,**B**) focal plane with 3D stacks showing co-localization (arrowheads) between BrdU (green) and GAD67 (red) in the IML of a control mouse of 60 days of survival; note that only BrdU-positive nuclei clearly surrounded by GAD67-positive cytoplasmic staining were counted, but not those nuclei that were partially flanked by GAD67 staining (arrow; see supplementary Material and Methods). (**C**,**D**) Charts showing the percentages of co-localization of both markers; no effect of the radiation was detected 15 days after BrdU injection (**C**), the differences appear 60 days after BrdU injection in all analyzed bulbar layers (**D**). *p < 0.05. Scale bar 20 μm.

First, we employed the glutamic acid decarboxylase of 67 kDa (GAD67) as an estimator of the differentiation to the GABAergic phenotype. We detected the co-localization of both GAD67 and BrdU in all the layers analyzed of both experimental groups at both survival times (Fig. 2; Supp. Fig. 5). Statistical analyses revealed no significant differences between experimental groups 15 days after BrdU injection (GL, p = 1.000; EPL, p = 1.000; IML, p = 0.222; Fig. 2C). However, at 60 days after BrdU injection, irradiated mice presented lower percentages of co-localization of both BrdU and GAD67 than controls in all of the layers analyzed (GL, p = 0.036; EPL, p = 0.036; IML, p = 0.036; Fig. 2D).

**Figure 3:**
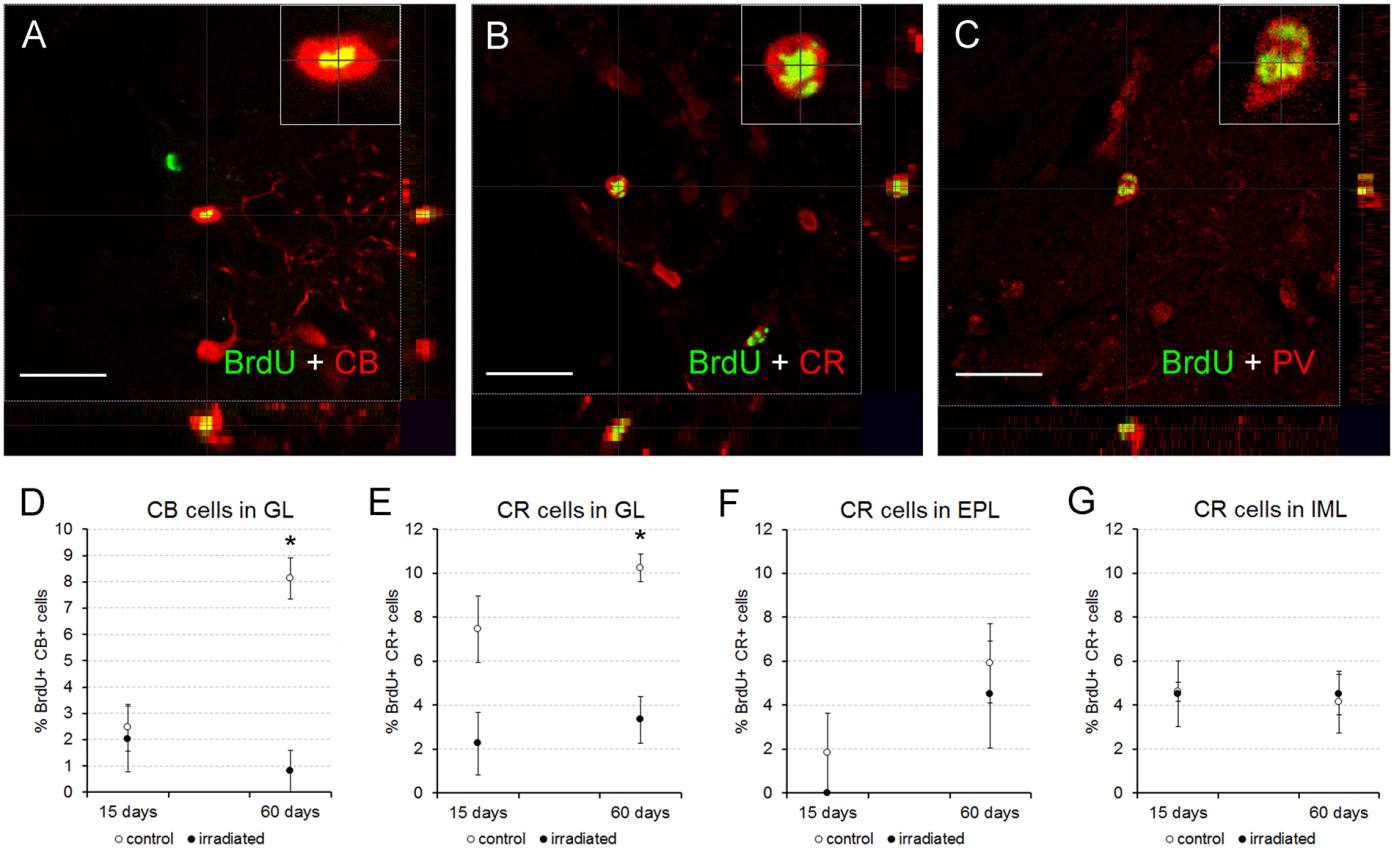
Cell characterization. (**A–C**) Focal planes with 3D stacks showing co-localization between BrdU (green) and calbindin (CB, red; **A**), calretinin (CR, red; **B**) or parvalbumin (PV, red; **C**); inset panels show a higher magnification of double-labelled cells; images correspond to control mice of 60 days of survival. (**D–G**) Charts showing the percentages of co-localization between BrdU and the other markers; differences for both CB (**D**) or CR (**E–G**) were focused on the glomerular layer 60 days after BrdU injections (**E**). *p < 0.05. Scale bar 20 μm.

Calcium-binding proteins determine well-defined neuronal populations in the OB^13, 32^; thus, we analyzed the differentiation of three of them: calbindin (CB), calretinin (CR) and parvalbumin (PV) positive neurons. CB-positive cells appeared mainly in GL (Supp. Fig. 5), being very scarce or nonexistent in other bulbar layers. CB and BrdU co-localization was detected in the GL, in both experimental groups at both survival ages (Fig. 3A,D). Surprisingly, we detected co-localization of CB and BrdU labeling in one cell in the IML of an irradiated mouse 60 days after BrdU injection (out of more than 200 cells analyzed; data not shown). The percentage of double-labelled BrdU and CB showed no differences between groups 15 days after BrdU injection (p = 0.690; Fig. 3D). Sixty days after BrdU injection, the percentage of newly generated cells that differentiated into CB interneurons increased in the control mice, contrary to what was observed in irradiated animals, demonstrating significant differences between both groups (p = 0.036; Fig. 3D).

The co-localization of CR and BrdU labeling was detected in all bulbar layers of both control and irradiated mice at both survival times (Fig. 3B,E,F,G; Supp. Fig. 5). The only exception to this result was that in the EPL 15 days after BrdU injection, no co-localization was detected in irradiated animals and only in some of the control mice (Fig. 3F). Irradiated mice presented a reduced percentage of double-labeled BrdU-CR cells than control animals, only in the GL 60 days after BrdU injection (for 15 days: GL, p = 0.056; EPL, p = 0.690; IML, p = 0.548; for 60 days: GL, p = 0.036; EPL, p = 0.571; IML, p = 0.786; Fig. 3E–G).

PV labelling appeared in GL and EPL (Fig. 3C; Supp. Fig. 5), and much more rarely in IML. PV-positive cells only co-localized with BrdU 60 days after injection. Statistical analysis revealed no significant differences in the percentage of co-localization between irradiated and control animals with respect to PV (GL, p = 0.393; EPL, p = 0.571; Supp. Fig. 6).

**Figure 4:**
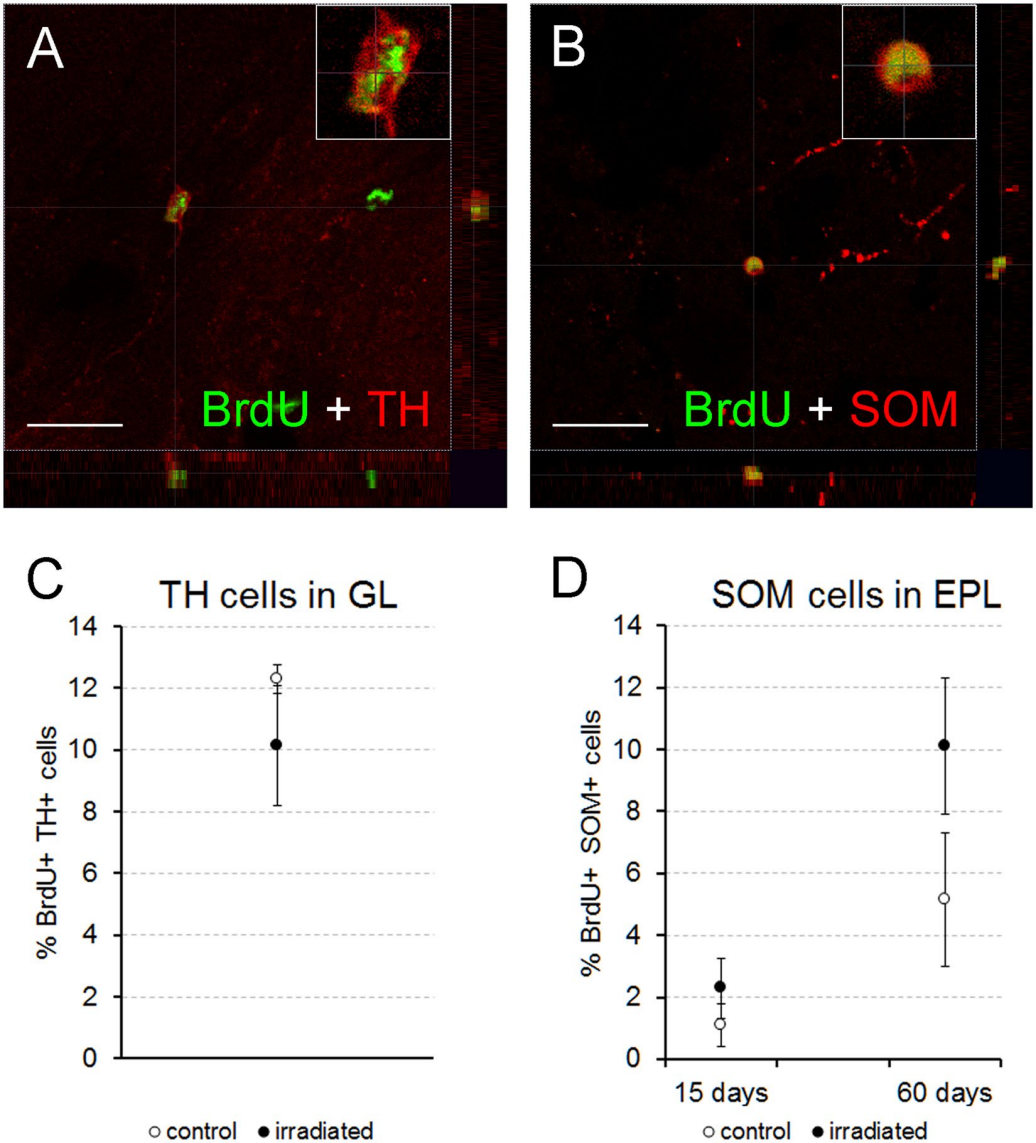
Cell characterization. (**A**,**B**) Focal planes with 3D stacks showing co-localization between BrdU (green) and tyrosine hydroxylase (TH, red; **A**) or somatostatin (SOM, red; **B**); inset panels show a higher magnification of double-labelled cells; images correspond to control mice of 60 days of survival. (**C**,**D**) Charts showing the percentages of co-localization of BrdU and the former markers; cells positives for both BrdU and TH appeared only in the GL 60 days after BrdU injection (**C**) BrdU-SOM positive cells were detected in the EPL (**D**) no differences were found between experimental groups for both markers at any survival time or bulbar layer. Scale bar 20 μm.

Tyrosine hydroxylase (TH) labeling (dopaminergic cells) was mainly detected in the GL (Fig. 4A; Supp. Fig. 5). TH interneurons have a late maturation^33^ and therefore its co-localization with BrdU was only observed 60 days after injection (Fig. 4C). The percentage of BrdU-TH double-labelled cells did not differ statistically between experimental groups (p = 0.571; Fig. 4C).

Finally, we analyzed the cells expressing somatostatin (SOM), since they constitute a population of interneurons considered important for bulbar odor discrimination^16^. Their expression was mainly restricted to the inner part of the EPL (Fig. 4B; Supp. Fig. 5), although scarce labeling was also found in the GL. We detected co-localization of SOM and BrdU in both experimental groups at both survival times in EPL. The percentage of this co-localization did not differ between irradiated and control animals (15 days, p = 0.421; 60 days, p = 0.571; Fig. 4D).

Taken together, these results demonstrate that radiation impairs not only the number of newly generated neuroblasts, but also their fate towards differentiation into specific neuronal populations, selectively affecting different populations of interneurons that modulate olfactory inputs whereas others remained unchanged.

### Radiation barely affects olfaction and bulbar electrophysiology

Since radiation affected bulbar neurogenesis and some neuronal populations of the OB, we decided to analyze both olfactory functionality and the bulbar physiology of irradiated animals, comparing them with controls.

**Figure 5:**
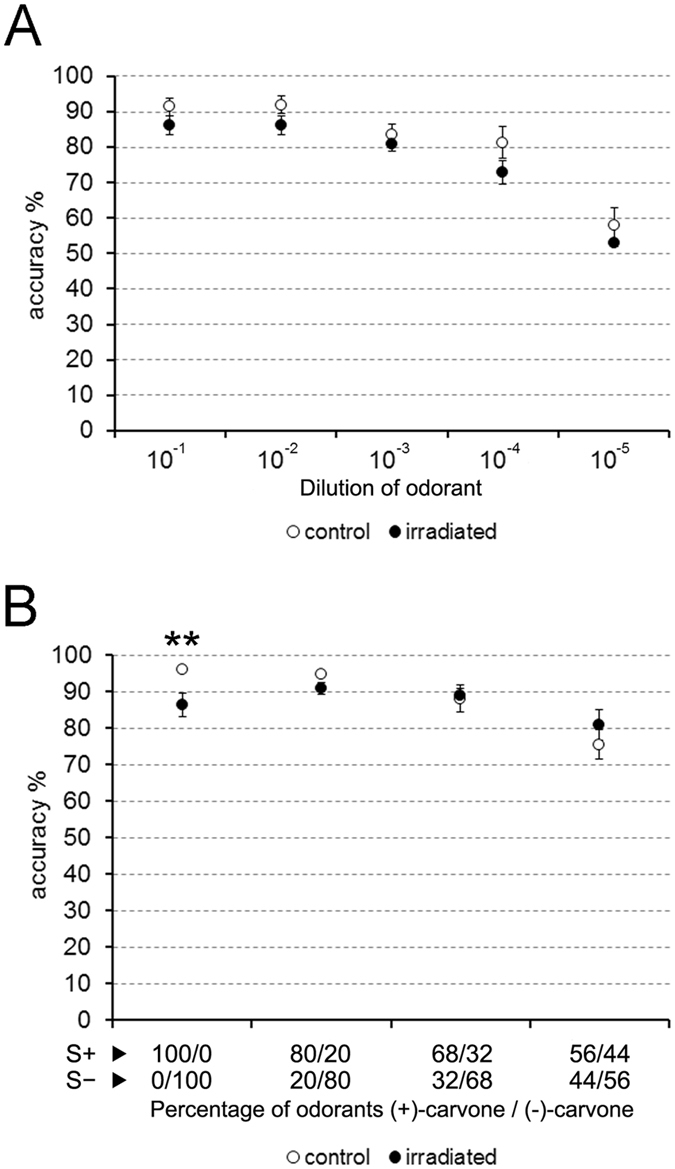
Olfactometry. (**A**) no differences between groups were observed in the detection test (carvone-(+)). (**B**) in the discrimination test, differences appeared only on the first experimental day (without the mixture of enantiomers carvone-(+) and carvone-(−)). **p < 0.01.

The olfactory functionality of mice was determined employing olfactometers. During the training period for the use of these devices, both experimental groups presented similar learning curves, reaching a plateau higher than 85% of accuracy on the same day (control, 91.5% ± 2.5 SEM; irradiated, 87.3% ± 2.6 SEM; p = 0.093; data not shown). After this initial stage, mice were subjected to detection and discrimination tests. First, our results revealed that both control and irradiated mice presented a similar detection threshold; they did not present statistical differences for detecting all the dilutions of carvone-(+) (the positive stimulus; S+) used in these experiments ([10^−1^], p = 0.093; [10^−2^], p = 0.069; [10^−3^], p = 0.529; [10^−4^], p = 0.129; [10^−5^], p = 0.771; Fig. 5A). Next, for the discrimination tests, a second odorant (the enantiomer carvone-(−)) was introduced as a negative stimulus(S−). Curiously, irradiated mice, even reaching a suitable accuracy of discrimination >85%, presented lower discrimination between stimuli than control animals in the first part of these tests (when both carvone enantiomers were still not mixed; p = 0.002; Fig. 5B). Conversely, in the subsequent discrimination tests (i.e. when both odorants were progressively mixed), statistical analyses did not reveal differences between the control and experimental groups (80/20, p = 0.093; 68/32, p = 0.539; 56/44, p = 0.283; Fig. 5B).

To analyze the electrophysiological functioning of the OB, we recorded local field potentials to measure gamma, beta and theta bands, which reflect the different OB network dynamics. In addition, these parameters were recorded in three different contexts that elicited different olfactory behaviors (immobility, exploration of a familiar environment and exploration of a new odorant).

**Figure 6:**
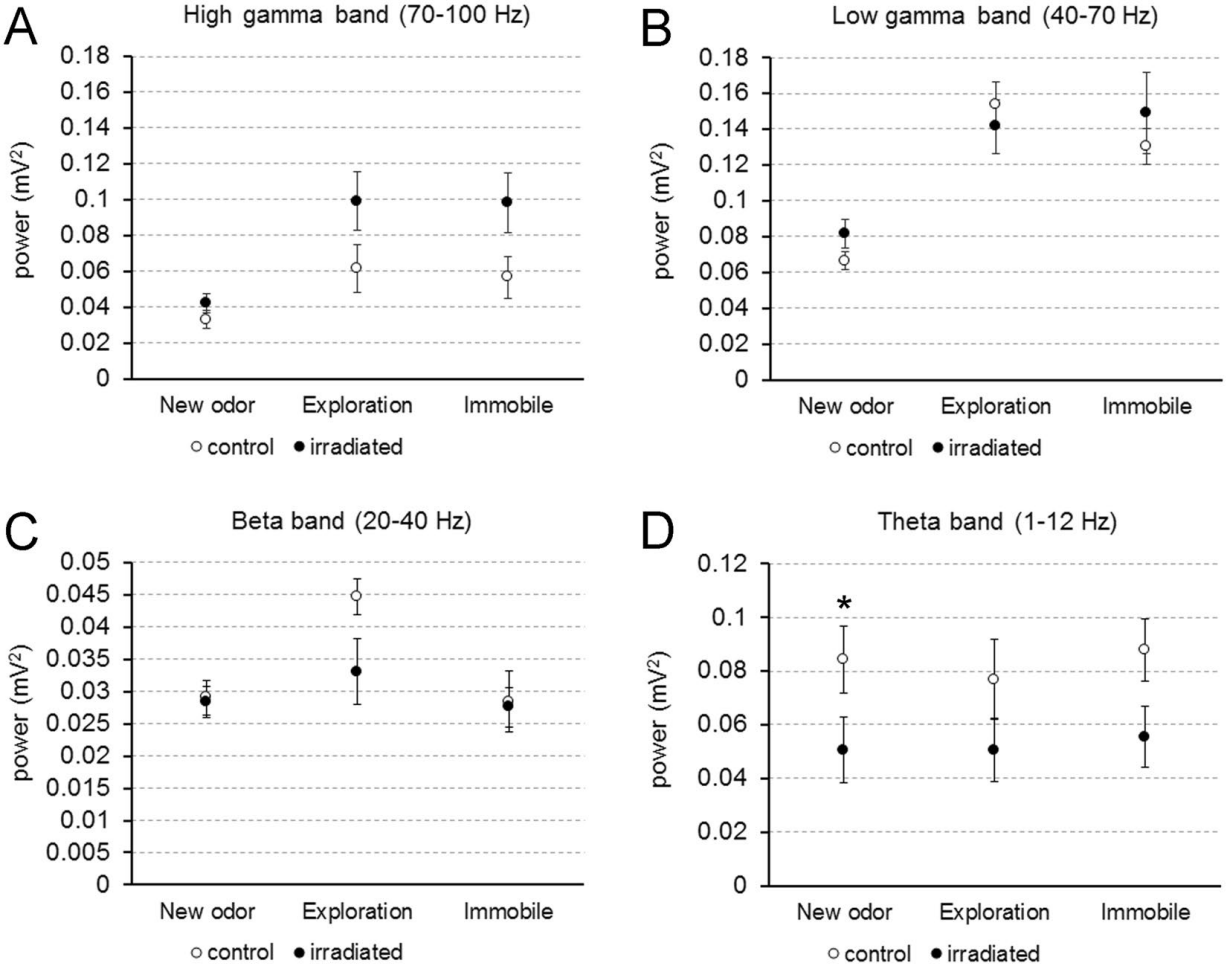
Recordings of local field potentials. (**A**,**B**) No statistical differences were found for the power of gamma oscillations (mitral cell firing) at any of the three behavioral contexts observed. (**C**) Beta oscillations (brain inputs and bulbar circuits) were also not affected by radiation. (**D**) Theta band (sniffing) was diminished in irradiated mice but only after the presentation of a new odorant. *p < 0.05.

Regarding gamma waves (40–100 Hz), our results demonstrated that irradiated animals usually had oscillations of an average higher amplitude than controls during exploration (control, 66.9 Hz ± 0.7 SEM; irradiated, 69.5 Hz ± 0.8 SEM; p = 0.049; data not shown). However, when the power of these gamma oscillations was analyzed, no significant differences were detected between experimental groups (new odor, p = 0.105; exploration, p = 0.574; immobile, p = 0.328), even when they were divided into low and high sub-bands (low gamma: new odor, p = 0.130; exploration, p = 0.574; immobile, p = 0.574; high gamma: new odor, p = 0.195; exploration, p = 0.083; immobile, p = 0.083; Fig. 6A,B). Similarly, the power of beta band (20–40 Hz) did not present differences between control and irradiated animals (new odor, p = 0.878; exploration, p = 0.105; immobile, p = 0.645; Fig. 6C). Therefore, it can be considered that the neuronal signals related to both gamma and beta oscillations were not affected by radiation. Conversely, the power of theta oscillations (1–12 Hz) presented a significant reduction in irradiated mice when a new odorant was presented (p = 0.049; Fig. 6D), although no additional differences were detected within the other behavioral contexts (exploration, p = 0.130; immobile, p = 0.083; Fig. 6D).

Altogether, these data indicated that almost all bulbar oscillations are largely unaffected after impairment in neurogenesis by high doses of radiation, and despite substantial alterations in the populations of interneurons in the OB. Only slight changes were detected in the theta wave (sniffing) when a new odorant was presented.

## Discussion

The OB is a structure that displays an important plasticity receiving olfactory axons, new neurons and showing adaptive changes of the centrifugal afferents and intrinsic neurons^34^. Using a multi-disciplinary approach, we have attempted to determine whether the OB was still capable of modifying its internal processes after severe damage in postnatal neurogenesis to overcome such impairment and ensure olfaction.

The number of newly-formed bulbar cells was significantly lower in irradiated than in control animals at both survival times, which agrees with previous results^28^. Concerning adult neurogenesis, the number of BrdU-labeled cells throughout time decreased in control mice, whereas it remained constant in irradiated animals (suggested in the latter as an escape from apoptosis compensating their reduced neurogenesis)^2, 8, 28^. Conversely, our results revealed an increase in BrdU labeling in control mice (from 15 to 60 days after BrdU injections) due to the high proliferation rate that characterizes postnatal neurogenesis^18, 19^. As the number of BrdU-labeled cells in irradiated mice was constant throughout time, radiation not only decreased cell proliferation, but in young mice (P19), also inhibited its increase. Hence, the damage in postnatal neurogenesis when the OB is still growing^18^ was much more dramatic than what was observed in adult animals; it compromised normal bulbar growth and resulted in a lower volume in comparison with controls (which can be appreciated with the naked eye at P300)^26^.

Concerning the differentiation of interneuron populations, the effects of radiation were more apparent 60 days after BrdU injections, once tangential migration and differentiation had taken place^8^. Moreover, our results demonstrated a relative alteration of the neuronal fate, especially in the GL (see below for further explanation). The populations of newly-formed interneurons positive for GAD67, CB or CR decreased, whereas PV- and SOM-immunopositive cells remained unaffected by radiation. Two different modes of bulbar neurogenesis have been defined: a constant net addition of newly generated interneurons in the GL *vs*. a minor fraction of cell turnover in the granule cell layer^20^. The rate of neurogenesis and cell turnover in the GL is constant from postnatal to adult stages^35^ whereas the rate of neurogenesis in the granule cell layer gradually decreases and becomes stabilized^20^, thus alterations within the GL would be particularly relevant.

The most common fate of neuroblasts in the OB is to become GABAergic interneurons^1, 2, 36^. Sixty days after injections the percentage of newly-formed interneurons expressing GAD67 decreased in all layers of the OB, possibly due to a delay in the differentiation of neuroblasts caused by radiation^37^. Another possibility may be a biochemical plasticity, since it has been reported that the expression pattern of GAD isoforms changes substantially after severe damage^38, 39^; thus, interneurons may use another mechanism for synthesizing GABA, including GAD65, which was not detected by the GAD67 antibody. Additionally, the reduction of GAD67 expression may be also due to a differentiation towards other subpopulations expressing other neuronal markers.

Additionally, our results at P60 demonstrated that the differentiation pattern of neuroblasts to GABAergic neurons in control animals exhibited a clear trend: a bigger percentage in the inner parts of the OB (i.e. IML) *vs*. a lower percentage in the outer ones (i.e. GL; Fig. 2D). This finding agrees with previous reports^1, 2, 6^. By contrast, this trend in differentiation was reversed in irradiated animals (Fig. 2D). Therefore, it is plausible to think that after radiation the differentiation into GABAergic neurons is prioritized within the GL *vs*. the IML. Since GL is more affected by the effects of radiation, this change in the differentiation of the GABAergic neurons may minimize these effects in order to preserve the functionality of the OB as much as possible. Conversely, the cellular and physiological features of the IML make this region more resistant to the impairments in cell differentiation, as is supported by the results of the electrophysiology experiments (see below).

Regarding the remaining markers, the changes elicited by radiation were circumscribed to the neuronal populations expressing CB or CR, in the GL, 60 days after BrdU injection. In mice, GL interneurons are classified into two populations: type 1, positive or negative to GABA; and type 2, always positive to GABA, and also positive for either CB or CR (at least to 65%)^40, 41^. According to our results, apart from reducing the number of new periglomerular neurons, radiation mainly impairs the differentiation to type 2 interneurons. Conversely, the differentiation rate to type 1 seems to be less affected by radiation, since these interneurons also co-express TH (particularly dopamine-GABAergic juxtaglomerular neurons)^36, 40, 42, 43^ whose final percentage did not change, and seem to be important for olfactory processing^44^. Particularly, the differentiation rate towards the small periglomerular subpopulation that co-localize PV and BrdU^45^ remained constant after radiation. Similarly, in the EPL no significant change was detected in the differentiation rates towards CR−, PV−, or SOM - positive cells.

These populations are essential for fine odor processing, especially those interneurons of the EPL expressing PV and SOM^16, 46, 47^. Thus, the preservation of the differentiation rate for these specific populations would help to maintain bulbar functionality. This seems to indicate that the system is flexible and is capable of altering the differentiation ratios in response to changes in proliferation, as previously suggested^23, 36^.

Also, it should be noted that our results do not allow us to discern if the changes in neuronal fate are triggered at the level of early/late progenitors or immature neurons, as there is a regionalization of different niches of neural precursor cells that give rise to different types of neuroblasts^12, 48^, and the different parts of the SVZ-RMS-OB axis have different regulation^49, 50^. In addition, the neurogenic brain regions have different resistance to ionizing radiations^51–53^. Accordingly, we cannot discard the possibility that different subpopulations of precursor/stem cells have different resistance to radiation. Therefore, radiation may affect certain types of precursor cells more than others, thus varying the proportion of the types of new interneurons in the olfactory bulb.

At this point, and although it is beyond the scope of this work, we also cannot discard differentiation towards glia, which should be due to progenitors of the OB parenchyma instead of the SVZ-RMS, which are mainly engaged to become interneurons^54^. Since very few proliferating cells were identified in the bulbar parenchyma in both irradiated and control mice^26^, the proportion of newly generated glial cells should be negligible in comparison with new interneurons. In parallel, we also cannot discard additional systemic side effects of the whole-body irradiation that might sharpen the slight impairments detected by both olfactometry and electrophysiology as explained below, or other compensatory mechanisms in other olfactory regions (i.e. secondary olfactory cortices).

Although we cannot completely reject further variations, the changes seen in interneuron populations could be considered somewhat stabilizing; a bulbar homeostasis is maintained as olfaction and physiology are almost unaffected by radiation damage. Concerning olfaction, its stability after severe radiation damage to postnatal neurogenesis is remarkable, which concurs with previous findings^27, 28^. Only slight impairment was detected in the discrimination tests, precisely in the first exposure to a new odorant. This divergence can be explained by differences between impairment in postnatal *vs*. adult neurogenesis, which is even more severe in the former, as explained above. In contrast, previous experiments employing other techniques for both neurogenesis disruption and olfaction assessment reported impairment in olfaction^55, 56^. Therefore, it might be hypothesized that the mechanisms aimed at the maintenance of a suitable olfaction depend on the nature and the timing of the damage and may not be universal for all odorants and all types of damage^44^.

Regarding electrophysiology, gamma and beta bands remained stable after radiation. Gamma oscillations reflect the activity between mitral and granule cells, and constitute an important dynamic of the olfactory information coding in the rodent brain^57, 58^. When projection neurons or their circuitry are damaged, gamma oscillations also become altered, thus reducing olfactory abilities^14, 27^. This agrees with our results, since radiation did not affect mitral cell density and, in general, olfactory abilities. There are two principal regions where the connections of mitral cells affect gamma oscillations: 1) the IML with the reciprocal synapses between mitral and granule cells^14, 57^; and 2) the EPL where SOM- and PV-positive interneurons also modulate gamma oscillations^16, 46, 47^. Disruption of neurogenesis probably has a relatively low impact in the huge population of granule cells: to detect changes in the gamma oscillations due to impairment in the deep bulbar circuitry, a significant population of granule cells must be altered, using either mutant animals or chemical antagonists^14, 57^. Conversely, the interneuron populations of the EPL are much smaller^32^ and possibly more sensitive to neurogenesis impairments. This could explain how the presence of a trend towards maintaining differentiation of new interneurons in the EPL could help OB homeostasis. Beta band is linked with a network between olfactory epithelium, OB and other olfactory cortices^59, 60^. There are several cues that may explain the stability of this band in irradiated mice. First, to our knowledge there are no previous works reporting radiation-related changes in brain central structures, apart from neurogenic niches^24, 61^. Therefore, this component of beta oscillations might not be affected. Moreover, impairment in the olfactory epithelium can be also discarded: no major alterations in this structure have been reported, even after applying very high radiation doses^62^. Additionally, beta oscillations are related with olfactory learning^59^ that was similar in all mice, in agreement with the stability of beta band in irradiated mice.

Theta oscillations can be mainly considered stable, although they were slightly affected by radiation. Theta oscillations are related to the bulbar activation caused by breathing and sniffing, and with the functioning of olfactory glomeruli^60, 63^. Once impairment in the olfactory epithelium is discarded, the change observed in this band could be explained by two findings: 1) by the reduction in size and cell recruitment of the OB, meaning when fewer cells are recruited, the currents and oscillation amplitude generated are also smaller; and 2) by an altered functioning of the olfactory glomeruli^63^, whose cell turnover is severely impaired by radiation. Finally, the only context in which theta oscillations were impaired in irradiated animals was in the exploration of a new odorant, which explains the isolated differences in olfactometry. It was observed that during the detection tasks only one odorant was employed as S+, but in the first session of the discrimination tasks (when differences appeared) mice operated with a second novel odorant as S−. Therefore, irradiated mice may exhibit slight difficulty in the discrimination of odors when a new odorant appears, which subsequently causes changes in the observed theta oscillations. Conversely, once the S− is recognized by successive exposures and is no longer a novel stimulus, these differences disappear. Additionally, the initial modulations of mitral cell activity by glomerular interneurons are important for odor discrimination^64^. Therefore, although gamma oscillations are not affected, impairment in neurogenesis in the GL could slightly alter the ability to discriminate odorants, especially in the case of similar molecules such as enantiomers.

In conclusion, the OB presents a remarkable plasticity, which allows severe damage to be overcome and leads to a rather intact functioning that permits odor detection and discrimination. Our study suggests that part of this plasticity may be obtained by varying the ratios of several immunohistochemically different populations of interneurons that adapt to new biological scenarios. This adaptation seems to, at least, boost GABAergic differentiation towards the outer layers of the OB, and maintain the fate of SOM-positive interneurons. These constitute a small but important population of cells, directly modulate mitral cell firing.

## Material and Methods

### Animals

Male mice of the C57BL/DBA strain (*Mus musculus*, L. 1758) were used to avoid the effect of female hormones^49^. The animals were housed at the Animal Facilities of the University of Salamanca or the Pasteur Institute at constant temperature and humidity, with a 12/12-hour photoperiod, and fed *ad libitum* with water and standard rodent chow. For all experiments two groups of animals were analyzed: irradiated and non-irradiated mice.

All animals were housed, manipulated and sacrificed in accordance with current European Legislation (2010/63/UE and Recommendation 2007/526/CE); the Bioethical Committee of the University of Salamanca approved this study.

### Irradiation of mice and bone marrow transplantation

Animals were irradiated at P19 with a ^137^Cs source (Gammacell 1000 Elite; MDS Nordion, Ottawa, Canada), with a dose of 7.5 Gy, strong enough to disrupt forebrain neurogenesis^26^. Twenty-four hours after irradiation, irradiated mice were transplanted with new bone marrow to ensure their survival. Mice of the same strain were used as donors, and the bone marrow cells were harvested as previously described^26^ (see supplementary material). 7.5 × 10^6^ cells were transplanted through the tail vein of each animal in a maximum volume of 150 µl of phosphate-buffered saline (PBS).

This protocol including the radiation dose allows the results of this work to be compared to results previously obtained in our laboratory^26, 27, 65^. Additionally, bone marrow transplants do not alter the ratio of cell proliferation^26^.

### Bromodeoxyuridine injections

To label proliferating cells, mice were administered with a mix of 5-bromo-2′-deoxyuridine (BrdU; 30 μg/g b.w.) and 5-fluoro-2′-deoxyuridine (FdU; 3 μg/g b.w.) in 0.1 M PBS, in three consecutive doses separated by three hours, each at P35. The survival after these injections was either 15 or 60 days, as previously described^8, 33^. Five animals were utilized for each group (control or irradiated) at each survival time.

### Olfactometry

To determine both the olfactory detection threshold and the olfactory discrimination of mice, custom-built, computer-controlled, six-channel air-dilution olfactometers were employed, based on previous models^66, 67^. The functioning of such devices was based on electronic valves controlling purified air streams, which were passed through mineral oil with diluted odorants (see supplementary material).

Ten irradiated and ten non-irradiated mice were utilized in these tests, starting at approximately P95. Partially water-deprived mice were trained to use these devices through an operant conditioning go/no-go paradigm (Supp. Fig. 2). Animals had to insert their snouts into the odor sampling port for at least 1.2 s and respond by licking a water delivery tube (within the same port) to receive a water reward (2 μl) in the presence of a positive odor stimulus, (+)-carvone (reinforced stimulus: S+; Supp. Fig. 2C). Additionally, the mice had to cease licking and retract their heads from the sampling port in the presence of an odorless air (unreinforced stimulus: S−; Supp. Fig. 2D). The percentage of correct responses (Supp. Fig. 2C–F) was determined in batches of 20 trials. In each trial, a single stimulus, S+ or S−, was presented in a random order. Each mouse underwent a session of 10 batches (200 trials) per day.

To determine the olfactory detection threshold of animals, decreasing concentrations of the odorant were used: [10^−1^], [10^−2^], [10^−3^], [10^−4^] and [10^−5^], testing each one per day. 24 hours after this evaluation, the olfactory discrimination of mice was analyzed; a new odorant was introduced, now having two odorants: (+)-carvone as S+ and (−)-carvone as S−, both diluted to [10^−2^]. First, these pure odorant molecules were presented to animals: only (+)-carvone for S+, and only (−)-carvone for S−. The following three days, we tested the discrimination threshold by presenting daily a binary odor mixture of the carvone enantiomers: 80–20% (80% (+)-carvone/20% (−)-carvone for S+, and 80% (−)-carvone/20% (+)-carvone the S−); 68–32% (68% (+)-carvone/32% (−)-carvone for S+, and 68% (−)-carvone/32% (+)-carvone the S−); and 56–44% (56% (+)-carvone/44% (−)-carvone for S+, and 56% (−)-carvone/44% (+)-carvone the S−).

### Electrophysiology

Eight mice of each group were used for this study and local field potential recordings were performed as previously described^14^ (see supplementary material). After surgery, a bipolar electrode (twisted 50 µm coated-platinum wires, impedance 0.2–0.5 MOhm) was placed into each of the two OB, with the tip of the bipolar electrode positioned into the lateral EPL (AP + 5.1 from Bregma, ML ± 1.45, DV −1.1 from brain surface). Additionally, two reference electrodes were also inserted into the occipital crest.

Seven days before the surgery, local field potentials were recorded in freely behaving mice. During the recordings, carried out in spontaneous exploration conditions, the mice remained in their home cages and the parameters of their behavior were continuously monitored. For spontaneous exploration of a new environment, a Petri dish with scented bedding (~20 µl of 1% amyl acetate in mineral oil, Sigma) was introduced into the cage. The continuous recordings were filtered and subjected to a Fast Fourier Transformation (Hanning-window, 2.44 Hz resolution) to obtain the spectral power and mean frequency of the beta (20–40 Hz), gamma (40–100 Hz), low gamma (40–70 Hz), high gamma (70–100 Hz), and theta (1–12 Hz) bands.

### Tissue preparation

Animals were deeply anaesthetized with 10 µl/g b.w. of chloral hydrate and perfused intracardially with 0.9% NaCl (w/v), followed by 5 ml/g b.w. of modified Somogyi’s fixative (with 4% paraformaldehyde w/v). After perfusion, the brains were dissected, postfixed for 2 hours in the same fixative, washed with PB and cryoprotected (30% w/v sucrose in PB). Forty-μm thick slices, were obtained using a freezing-sliding microtome attached to a freezing unit, were ordered in 6 series, and rinsed in PB (3 × 10 min).

### Cell labeling

BrdU-labelled cells were detected by immunohistochemistry as previously described^8, 33^. Briefly, after denaturing the DNA (incubation of 1 hour at 37 °C in HCl 2 N), sections were subjected to a standard protocol of immunelabeling revealed with 3,3′-diaminobenzidine (see supplementary material). The antibodies used were an anti-BrdU monoclonal rat primary antibody (1:5,000; Abcam, Cambridge, UK) and a biotinylated goat anti-rat secondary antibody (1:300; Jackson, West Grove, PA, USA).

For determining the neuronal fate of BrdU positive cells a double immunofluorescent technique was carried out (see supplementary material). In each reaction the anti-BrdU monoclonal rat antibody (1:5,000; Abcam) was combined with either rabbit anti-calbindin D-28k (1:2,000; Swant, Bellinzona Switzerland), rabbit anti-calretinin (1:2,000; Swant), rabbit anti-GAD67 (1:1,000; Merck-Millipore, Darmstadt, Germany), rabbit anti-parvalbumin (1:2,000; Swant), mouse anti-reelin (1:1,000; Millipore, Temecula CA, USA), rabbit anti-somatostatin (1:3,000; Swant) or mouse anti-tyrosine hydroxylase (1:10,000; Jacques Boy, Reims, France) antibodies. The correspondent secondary fluorescent antibodies were Cy2-conjugated goat anti-rat antibody (for BrdU-labelled cells; 1:500; Jackson), and other Cy3-conjugated goat antibodies (for the other antigens studied; 1:500; Jackson).

### Cell count and statistical analyses

For estimating the number of BrdU-positive cells, a one-in-six series of slices for each animal were analyzed. Comparative levels of each series were analyzed, according to previous studies^68, 69^ (see supplementary material). In each section, cells were counted in three different regions depending on the layering of the OB, as previously described^8^: GL, EPL and IML. The area of each region was measured with the Neurolucida (V8.23) and Neuroexplorer programs (V4.70.3), and used to estimate their volume employing the Table Curve 2D program (V5.0.1). Afterwards, 10 randomized images were taken in the different layers of the OB with an Olympus DP70 digital camera. The ImageJ program (V1.47) was used to estimate the mean volumetric density of BrdU-positive cells in each layer. Finally, this volumetric density was referred to the estimated volume to calculate the total number of BrdU-positive cells.

To determine the cellular fate of BrdU-labeled neurons, 5 areas were chosen at random for each animal and each bulbar layer of study, and analyzed with a confocal microscope (see supplementary material). Co-localizations were counted and represented as a percentage of the total BrdU-positive cells for each bulbar layer. Finally, the mean percentages of the 5 areas for the different bulbar layers were calculated.

All counts were performed by the same person (D. D.), following the same criteria and unbiased by a double-blind study (R. M-C. and E. W.).

The non-parametric Mann-Whitney U test was used to analyze possible differences between the irradiated and non-irradiated animals for each experiment. For all the statistical analyses, SPSS statistical package (V22) was used.

## Electronic supplementary material

Supplementary material
