# Supplementary material for: Olfactory bulb plasticity ensures proper olfaction after severe impairment in postnatal neurogenesis
Source: Sci Rep. 2017 Jul 18;7:5654. doi: 10.1038/s41598-017-05970-1 (PMC5516035; doi:10.1038/s41598-017-05970-1)
Supplement: Supplementary file 1 — Supplementary material [file 41598_2017_5970_MOESM1_ESM.pdf]

- Supplementary material -

Olfactory bulb plasticity ensures proper olfaction after severe impairment in postnatal neurogenesis

Authors' names and institutions:

Díaz D. [1,2], Muñoz-Castañeda R. [1,2], Ávila C. [2,3], Carretero J. [2,4], Alonso J.R. [1,2,5], Weruaga E. [1,2]

1 Laboratory of Neuronal Plasticity and Neurorepair, Institute for Neuroscience of Castile & Leon (INCyL), Universidad de Salamanca, Salamanca, Spain.

2 Institute of Biomedical Research of Salamanca, IBSAL.

3 Applied Statistics Group, Department of Statistics, Universidad de Salamanca, Salamanca, Spain.

4 Department of Human Anatomy and Histology, Faculty of Medicine, University of Salamanca, Salamanca, Spain

5 Instituto de Alta Investigación, Universidad de Tarapacá, Arica, Chile.

Extended material and methods

Animals

Male mice of the C57BL/DBA strain (*Mus musculus*, L. 1758) were used in the experiments. The use of females was avoided because oestral hormonal stages affect neurogenesis in the forebrain (Díaz et al., 2009). The animals were housed at the Animal Facilities of the University of Salamanca or the Pasteur Institute at constant temperature and humidity, with a 12/12 hour photoperiod, and fed *ad libitum* with water and standard rodent chow. For all experiments two groups of animals were analyzed: irradiated and non-irradiated mice. The former received bone marrow transplants to ensure their survival after irradiation (see below). Adult mice of about P60 of the same strain were utilized as donors (see below).

All animals were housed, manipulated and sacrificed in accordance with current European Legislation (2010/63/UE and Recommendation 2007/526/CE) and the Bioethical Committee of the University of Salamanca approved this study.

Irradiation of mice and bone marrow transplantation

Animals were irradiated at P19 (Supp. Fig. 1) with a gamma irradiation device with a ^{137}Cs source for mice, model Gammacell 1000 Elite (MDS Nordion, Ottawa, Canada). This device provides a radiation rate of 243 cGy/min, with energy of 0.662 MeV. The radiation dose chosen was 7.5 Gy (minimal lethal dose), strong enough to disrupt forebrain neurogenesis (Díaz et al., 2011).

As radiation also ablates the bone marrow of mice, twenty-four hours after irradiation (Supp. Fig. 1), mice were transplanted with new bone marrow to ensure their survival (at P20). Mice of the same strain were used as donors as previously described (Díaz et al., 2012). Bone marrow extraction was carried out by injecting IMDM medium (Iscove´s Modified Dulbecco´s Medium; Invitrogen; Carlsbad, CA, USA) with an insulin syringe at both epiphyses of femurs and tibias. The bone marrow wash was filtered through a 70-µm pore size filter (BD Falcon; Bedford, MA, USA) and centrifuged at 1500 rpm for 5 min. The supernatant was removed and

the pellet was resuspended in 5 ml of lysis buffer (140 mM NH_4Cl, 17 mM Tris-base, pH 7.4) for 5 min to break up the erythrocytes. The reaction was stopped by adding 45 ml of 0.1 M phosphate-buffered saline, pH 7.4 (PBS). An aliquot was collected to estimate the number of BMSC extracted using a Thoma chamber, meanwhile the cell suspension was centrifuged again at 1500 rpm for 5 min and the pellet was resuspended in PBS. 7.5 x 10^6 cells were transplanted through the tail vein of each animal in a maximum volume of 150 µl. The volume of the cell suspension injected into each mouse was adjusted depending on the concentration of cells, in order to inject the same number of cells into all animals.

Apart from severely impairing postnatal neurogenesis, this protocol including the radiation dose also allows the results of this work to be compared to results previously obtained in our laboratory (Díaz et al., 2011; Recio et al., 2011; Díaz et al., 2012). Additionally, it is necessary to remark that only irradiated mice received bone marrow transplant, since the aim of this procedure was to allow the survival after the radiation, and we have discarded the influence of bone marrow transplants on the SVZ (Díaz et al., 2011), i.e. bone marrow transplants do not alter the ratio of cell proliferation.

After the irradiation and transplant, animals were housed separately in an insulated rack specific for immunodepressed animals at the Animal Facilities of the University of Salamanca for two weeks and thereafter under standard conditions (Supp. Fig. 1).

Bromodeoxyuridine injections

The thymidine analog 5-bromo-2’-deoxyuridine (BrdU; Sigma-Aldrich, Darmstadt, Germany; 30 µg/g b.w.) was administered to both irradiated and control mice, mixed with the thymidine synthesis inhibitor 5-fluoro-2’-deoxyuridine (FdU; Sigma-Aldrich; 3 µg/g b.w.) in 0.1 M PBS, to label proliferating cells. The BrdU/FdU (10:1 v/v) mixture was injected intraperitoneally in three consecutive doses separated by three hours, each at P35 (24 hours after the quarantine in the insulated rack, in the case of irradiated mice). The survival times applied after these injections were 15 days (sacrifice at P50) and 60 days (sacrifice at P95; Supp. Fig. 1),

to study the migration of neuroblasts and the survival of newly generated neurons respectively, as previously described (Valero et al., 2007; Curto et al., 2014). Five animals were utilized for each group (control or irradiated) at each survival time (15 or 60 days).

Olfactometry

To determine both the olfactory detection threshold and the olfactory discrimination of mice, custom-built, computer-controlled, six-channel air-dilution olfactometers were employed, based on previous models (Bodyak and Slotnick, 1999; García-González et al., 2016). The functioning of such devices was based on electronic valves controlling purified air streams, which were passed through mineral oil with diluted odorants. The odorized air was diluted again 1:40 in another odor-free air stream before being introduced into an odor sampling port attached to a mouse operant chamber. The odorants used were the enantiomers (+)-carvone (96 %; Sigma-Aldrich) and (−)-carvone (98 %; Sigma-Aldrich).

Ten irradiated and ten non-irradiated new mice were utilized in these tests. Olfactometry experiments started around P95, that is ~75 days after the radiation (in its case), and coinciding with the longer survival time of BrdU injected mice (Supp. Fig. 1). Partially water-deprived mice (80–85 % of their baseline body weight) were trained to use these devices through an operant conditioning go/no-go paradigm (Supp. fig. 2). Following a standard operant conditioning method, animals had to insert their snouts into the odor sampling port for at least 1.2 s and respond by licking a water delivery tube (within the same port) to receive a water reward (2 μl) in the presence of a positive odor stimulus, (+)-carvone (reinforced stimulus: S+; Supp. Fig. 2C). Additionally, the mice had to cease licking and retract their heads from the sampling port in the presence of an odorless air (unreinforced stimulus: S−; Supp. Fig. 2D). The percentage of correct responses (Supp. Fig. 2C-F) was determined in batches of 20 trials. In each trial, a single stimulus was presented and S+ and S− trials were presented in a random order. Each mouse underwent a session of 10 batches (200 trials) per day. The accuracy of each session was calculated as the mean score of the 7 last batches (more constant), thus discarding the first three. This was done to avoid any biased results that might have occurred at the onset of the execution of the daily task.

Mice were first trained with (+)-carvone versus mineral oil, reaching a plateau of performance (with an accuracy of minimum 85 %) after four training sessions. Then, to determine the olfactory detection threshold of animals, decreasing concentrations of the odorant were used: [10^{-1}], [10^{-2}], [10^{-3}], [10^{-4}] and [10^{-5}], testing each one per day. 24 hours after this evaluation, the olfactory discrimination of mice was analyzed. For this test a new odorant was introduced, now having two odorants: (+)-carvone as S+ and (−)-carvone as S−, both diluted to [10^{-2}]. The first day of the discrimination tests, these pure odorant molecules were presented to animals: only (+)-carvone for S+, and only (−)-carvone for S−. The following three days, we tested the discrimination threshold by presenting daily a binary odor mixture of the carvone enantiomers: 80-20 % (80 % (+)-carvone/20 % (−)-carvone for S+, and 80 % (−)-carvone/20 % (+)-carvone the S−); 68-32 % (68 % (+)-carvone/32 % (−)-carvone for S+, and 68 % (−)-carvone/32 % (+)-carvone the S−); and 56-44 % (56 % (+)-carvone/44 % (−)-carvone for S+, and 56 % (−)-carvone/44 % (+)-carvone the S−).

Electrophysiology

Eight mice of each group were used for this study after olfactometry tests. The mice were the same employed for olfactometry, excluding those animals weakened or dead by the surgery of this procedure (Supp. Fig. 1). Electrophysiological recordings were performed as previously described (Lepousez and Lledo, 2013). Briefly, animals were deeply anaesthetized with ketamine/xylazine (intraperitoneal injections of 100/10 mg/kg, i.p.; Imalgene/Rompun, Merial and Bayer, Lyon, France). Then, the skull was exposed and a bipolar electrode (twisted 50 µm coated-platinum wires, impedance 0.2-0.5 MOhm, A-M systems, WA, USA) was placed into each of the two olfactory bulbs (OB), with the tip of the bipolar electrode positioned into the lateral external plexiform layer (EPL; AP +5.1 from Bregma, ML ±1.45, DV -1.1 from brain surface). Two reference electrodes were also inserted into the occipital crest. All electrodes were fixed to the skull with a liquid bonding resin (Superbond, Sun Medical, NJ, USA) and dental acrylic resin (Unifast, Sun Medical). The animals were administered ibuprofen as an analgesic and left in their cages to recover for 7 days before the start of the recordings.

Local field potential recordings were amplified (x1000, 0-300 Hz, WPI, Hertfordshire, UK) and sampled (Micro1401-3 A/D interface, CED, Cambridge, UK) at 5 kHz, in freely behaving mice. During the recordings, carried out in spontaneous exploration conditions, the mice remained in their home cages and the parameters of their behavior were continuously monitored. For spontaneous exploration of a new environment, a Petri dish with scented bedding (~20 µl of 1% amyl acetate in mineral oil, Sigma-Aldrich) was introduced into the cage. Local field potential signals (5 kHz) from each hemisphere (two recordings for each mouse) were band-pass filtered (FIR filter, 1-300 Hz) and 1-min-long epochs of the signal were extracted every 10 min along the continuous recordings and subjected to a Fast Fourier Transformation (Hanning-window, 2.44 Hz resolution) to obtain the spectral power and mean frequency of the beta (20-40 Hz), gamma (40-100 Hz), low gamma (40-70 Hz), high gamma (70-100 Hz), and theta (1-12 Hz) bands. Absolute power values were also normalized to the total power of the spectrum.

Tissue preparation

Animals were deeply anaesthetized with 10 µl/g b.w. of chloral hydrate (Prolabo, Fontenay-sous-Bois, France) and perfused intracardially with 0.9 % NaCl (w/v), followed by 5 ml/g b.w. of modified Somogyi's fixative (4 % w/v of depolymerized paraformaldehyde and 15 % v/v saturated picric acid in 0.1 M phosphate buffer, PB). After perfusion, the brains were dissected, postfixed for 2 hours in the same fixative and washed thoroughly in PB.

Tissue blocks were cryoprotected by immersing them in 30 % (w/v) sucrose in PB. When the blocks had sunk, they were frozen with liquid nitrogen and stored at -80 ºC until sectioning. Forty-µm thick slices, obtained using a freezing-sliding microtome (Jung SM 2000, Leica Instruments, Nussloch, Germany) attached to a freezing unit (Frigomobil, Leica Instruments), were ordered in 6 series, and rinsed in PB (3 x 10 min).

Cell labeling

Immunohistochemistry techniques were performed to detect the cells labeled with BrdU and to estimate the number of cells present at both survival times (15 and 60 days after BrdU

injection). Tissue slices were incubated for 1 hour at 37 ºC under continuous rotary shaking in HCl 2 N to denature the DNA and ensure the access of the antibodies to the BrdU. Afterwards, the slices were rinsed in 0.1 M borate buffer (pH 8.5; 3 x 10 min) and then in PBS (3 x 10 min). Sections were incubated with a medium containing 0.2 % (w/v) Triton X-100, 5 % (v/v) normal goat serum, and the primary anti-BrdU monoclonal rat antibody (1:5,000; Abcam, Cambridge, UK) in PBS, during 72 h at 4 ºC under continuous rotary shaking. Following this, sections were washed in PBS (3 x 10 min) to remove the unbound antibody, and incubated for 2 hours at room temperature under continuous rotary shaking with a biotinylated goat anti-rat antibody (1:300; Jackson, West Grove, PA, USA) in PBS. Tissues were rinsed again in PBS (3 x 10 min) and incubated with avidin-biotin-peroxidase complex (Kit Elite ABC; 1:200; Vector laboratories, Burlingame, CA, USA) in PBS for 90 min at room temperature. Later, sections were rinsed in PBS (3 x 5 min) and 0.1 M Tris-HCl buffer (pH 7.6, 2 x 5 min). Finally, the labeling was visualized by incubating the sections in 0.02 % 3,3'-diaminobenzidine (w/v) and 0.003 % hydrogen peroxide (v/v) in 0.1 M Tris-HCl buffer. The reaction was controlled under the microscope and stopped with cold buffer. Sections were mounted on microscope slides, dehydrated using an increasing ethanol series and xylene and coverslipped with Entellan (Merck, Darmstadt, Germany).

For determining the neuronal fate of BrdU positive cells a double immunofluorescent technique was carried out. After DNA denaturation with HCl (see above), sections were incubated in a medium containing 0.2 % (w/v) Triton X-100, 5 % (v/v) normal goat serum and the primary antibodies in PBS, during 72 h at 4 ºC under continuous rotary shaking. In each reaction anti-BrdU monoclonal rat antibody (1:5,000; Abcam) was combined with either rabbit anti-calbindin D-28k (CB; 1:2,000; Swant, Bellinzona Switzerland), rabbit anti-calretinin (CR; 1:2,000; Swant), rabbit anti-glutamic acid decarboxylase 67 kDa (GAD67; 1:1,000; Merck-Millipore, Darmstadt, Germany), rabbit anti-parvalbumin (PV; 1:2,000; Swant), mouse anti-reelin (1:1,000; Millipore, Temecula CA, USA), rabbit anti-somatostatin (SOM, 1:3,000; Swant, Bellinzona Switzerland) or mouse anti-tyrosine hydroxylase (TH; 1:10,000; Jacques Boy, Reims, France) antibodies. After these incubations, the sections were washed in PBS (3 x 10 min) and incubated in a second

medium for 2 hours at room temperature under continuous rotary shaking. This second medium contained the correspondent secondary fluorescent antibodies in PBS: Cy2-conjugated goat anti-rat antibody (1:500; Jackson, West Grove, PA, USA) to label BrdU cells, and other Cy3-conjugated goat antibodies to label the correspondent antigens studied (1:500; Jackson). Slices were rinsed in PB (2 x 5 min), mounted and coverslipped with a home-made anti-fading medium made up by 0.42 % (w/v) glycine, 0.021 % (w/v) NaOH, 0.51 % (w/v) NaCl, 5 % (w/v) N-propyl-gallate, 70 % (v/v) glycerol and 0.002 % Thimerosal (w/v).

Cell count and statistical analyses

For estimating the number of BrdU-positive cells, a one-in-six series of slices for each animal were analyzed. First, the limits of the region of analysis in the OB were defined from Bregma 3.60 mm to Bregma 5.20 mm (Hof et al., 2000), thus ensuring the presence of all the bulbar layers without the presence of the accessory olfactory bulb, using comparative levels according to previous studies (Weruaga et al., 1999). In all of these selected sections, cells were counted in three different regions depending on the layering of the OB, as previously described for experiments comprising BrdU injections (Valero et al., 2007): glomerular layer (GL), EPL and the inframitral layers (IML), including both inner plexiform and granule cell layers (Valero et al., 2007). The area of each region was measured with the Neurolucida (V8.23, MicroBrightField, Colchester, VT, USA) and Neuroexplorer programs (V4.70.3, MicroBrightField). Then, these areas were used to estimate the volume of the three different layers in the bulbar region of study (as well as the total volume of such region), employing the Cavalieri method with the Table Curve 2D program (V5.0.1 SYSTAT Software, Chicago, IL, USA). Afterwards, 10 randomized images (a double-blind system was used for designate both the tissue slice and the location) were taken in the different layers of the OB for each animal with an Olympus DP70 digital camera (Olympus Optical, Tokyo, Japan) attached to an Olympus Provis AX70 photomicroscope (Olympus Optical). The ImageJ program (V1.47, Wayne Rasband, National Institutes of Health, USA) was used to estimate the mean volumetric density of BrdU-

positive cells in each layer. Finally, this volumetric density was referred to the estimated volume to calculate the total number of BrdU-positive cells in each bulbar layer of the analyzed region.

To determine the cellular fate of BrdU-labeled neurons, 5 areas were chosen at random (as described above) for each animal and each bulbar layer of study (always corresponding to the region of study) and analyzed with a confocal microscope (Leica TCS SP2, DMIRE2, Mannheim, Germany). In each of these areas, 8-10 focal planes were obtained to perform a 3D stack in order to detect the neuronal markers that co-localized with BrdU-positive cells. Focal planes were gathered with an optimized pinhole opening to ensure a correct detection of co-localization of markers. A special care was necessary for the quantification of double positive neurons for BrdU and GAD67, since the GAD67 staining is present in both cell body and processes. Therefore, after performing the 3D reconstruction stack, only those BrdU-positive nuclei that were closely surrounded by an intense and continuous ring of GAD67 staining were taken into account. The aim was to ensure the quantification of only those cells that are expressing the marker, thus avoiding other cells that are being contacted by GAD67-positive processes.

After quantification, the number of co-localizations was represented as the percentage of the total BrdU-positive cells for each bulbar layer. Finally, the values of co-localizations in each animal were obtained by calculating the mean percentages of the 5 areas for the different bulbar layers.

All counts were performed by the same person (D. D.), following the same criteria and unbiased by a double blind study (R. M-C. and E. W.).

The Kolmogorov-Smirnov test demonstrated the homoscedasticity (quality of samples with normal distribution and equal variances) for all of the studies. The non-parametric Mann-Whitney U test was used to analyze possible differences between the irradiated and non-irradiated animals for each experiment. For all the statistical analyses, SPSS statistical package (V22; IBM, NY, USA) was used.

Supplementary figure legends

Supplementary Fig. 1. Timeline of the experimental procedures.

Supplementary Fig. 2. Schema of the functioning of olfactometers. **A**; mice inserts its snout in the odor sampling port, breaking a laser beam and activating the system. **B**; once the laser beam is broken, odorized or odorless air comes into the odor sampling port and the mouse decides whether to lick the reward tube for water or not. **C-F**; the four possible mouse responses depending on the stimulus presented. **C**; when the stimulus is positive (S+) and the mouse licks the water delivery tube, it receives 2 µl of water as a reward and also scores a “hit” (correct response). **D**; when the stimulus is negative (S-), the mouse has to retract its head to score a “correct retreat” (correct response). **E**; the mouse incorrectly licks the water delivery tube in the presence of a negative stimulus (S-), called “false alarm”. **F**; the mouse retracts its head in the presence of a positive stimulus (S+), thus making a “miss”. Both “false alarm” and “miss” are incorrect responses that do not count when calculating the accuracy of mice during tests.

Supplementary Fig. 3. Effect of radiation OB volume. Images of comparable sections of control (**A**) and irradiated (**B**) mice; note the evident reduction of size in the latter. **C**, charts showing the volume reduction in the layers of the OB. M, medial; D, dorsal. Scale bar 500 µm. **, $p < 0.01$.

Supplementary Fig. 4. Effect of radiation in cell turnover. Comparison of the estimated number of BrdU-positive cells between the two survival times. Control mice present an increase of newly formed cells from 15 to 60 days after BrdU injection considering IML (**A**), GL (**C**) and the whole OB (**D**), whereas irradiated animals have a similar number of labelled cells in the same regions, 15 and 60 days after BrdU injection. Conversely, in the EPL (**B**) the number of BrdU-positive cells remained constant in control mice but decreased in irradiated animals. *, $p < 0.05$.

Supplementary Fig. 5. Cell characterization. Epifluorescence images showing the general staining for the different markers employed in both control (**A**,**C**,**E**,**G**,**I**,**K**,**M**,**O**,**Q**) and irradiated animals (**B**,**D**,**F**,**H**,**J**,**L**,**N**,**P**,**R**). Images were taken in those survival ages at which double staining for both BrdU (green) and the different markers (red) was found. **A-D** (staining for GAD67), **E-H** (staining for CB), **M-N** (staining for PV) and **O-P** (staining for TH) are focused in GL; **I-L**

(CR labelling) show all the analyzed bulbar layers; **Q** and **R** correspond to SOM staining and are focused in the EPL. Scale bar 100 μm for **A-H**; 200 μm for **I-L**; 100 μm for **M-P**; 100 μm for **Q-R**.

Supplementary Fig. 6. Chart showing the percentages of co-localization of BrdU and PV 60 days after BrdU injection; no differences we detected amongst control and irradiated mice; note that in the EPL the co-localization was very scarce, even inexistent in some subjects.

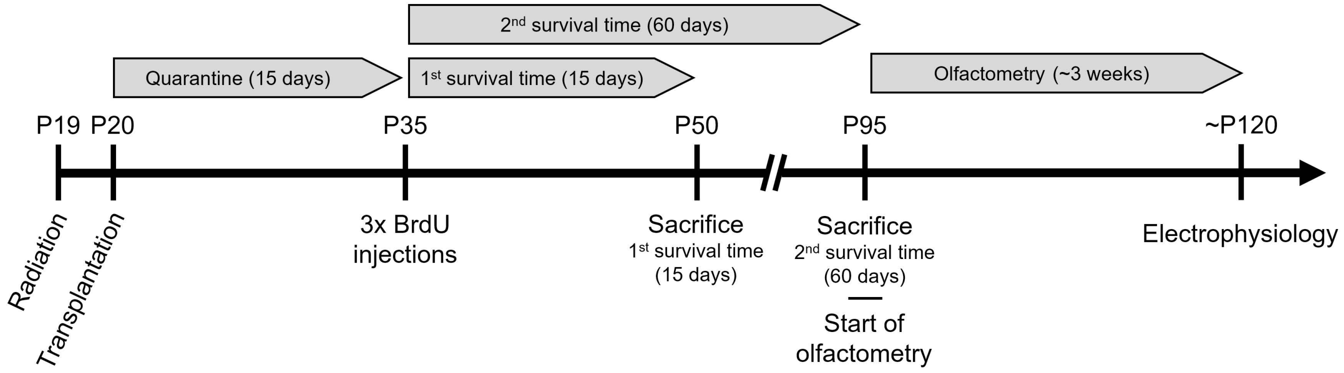

2nd survival time (60 days)
Quarantine (15 days)
1st survival time (15 days)
Olfactometry (~3 weeks)
P19
P20
P35
P50
P95
~P120
Radiation
Transplantation
3x BrdU injections
Sacrifice
1st survival time (15 days)
Sacrifice
2nd survival time (60 days)
Start of olfactometry
Electrophysiology

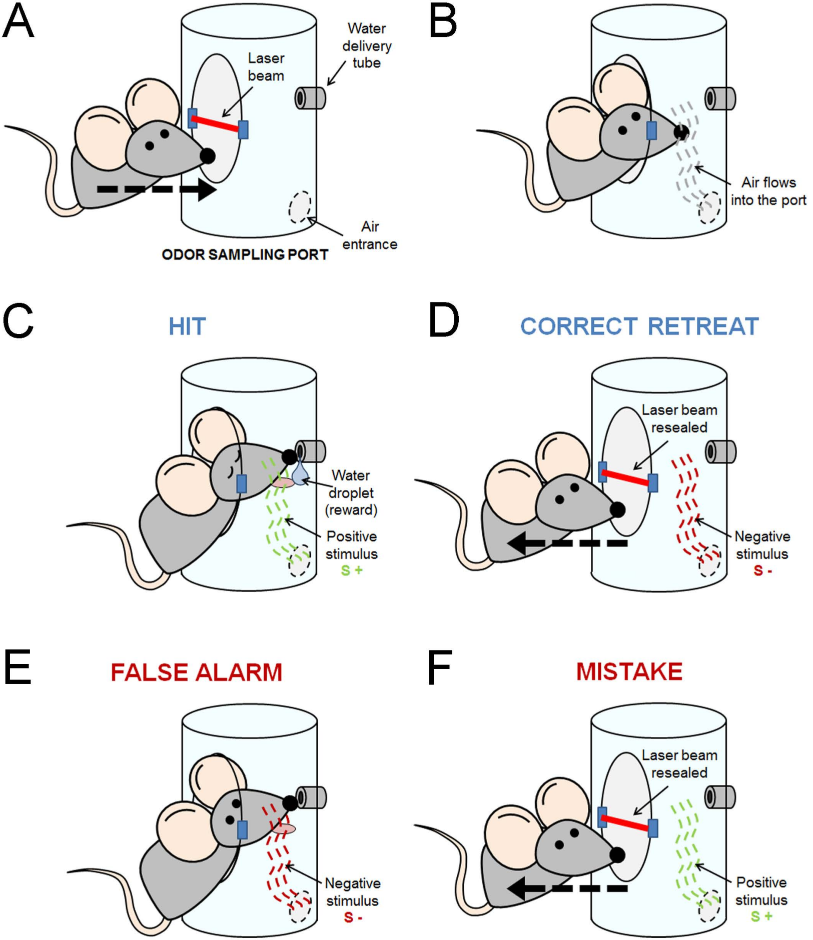

A
Water delivery tube
Laser beam
Air entrance
ODOR SAMPLING PORT
B
Air flows into the port
C
HIT
Water droplet (reward)
Positive stimulus
S +
D
CORRECT RETREAT
Laser beam resealed
Negative stimulus
S -
E
FALSE ALARM
Negative stimulus
S -
F
MISTAKE
Laser beam resealed
Positive stimulus
S +

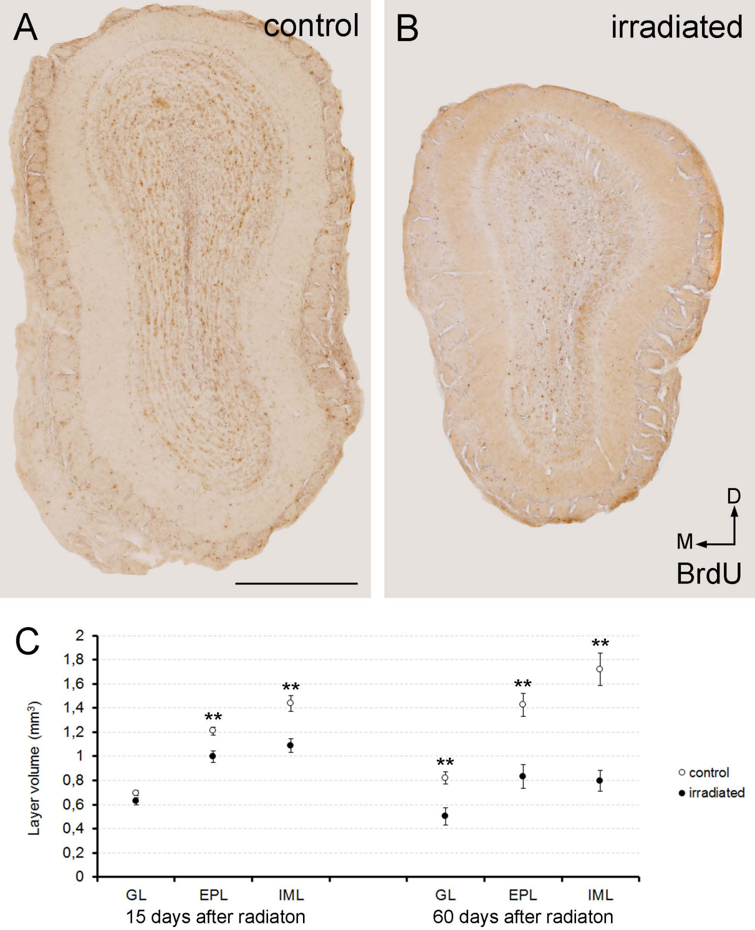
A
control
B
irradiated
D
M
BrdU
C
Layout volume (mm3)
2
1,8
1,6
1,4
1,2
1
0,8
0,6
0,4
0,2
0
**
GL
EPL
IML
15 days after radiaton
60 days after radiaton
control
irradiated

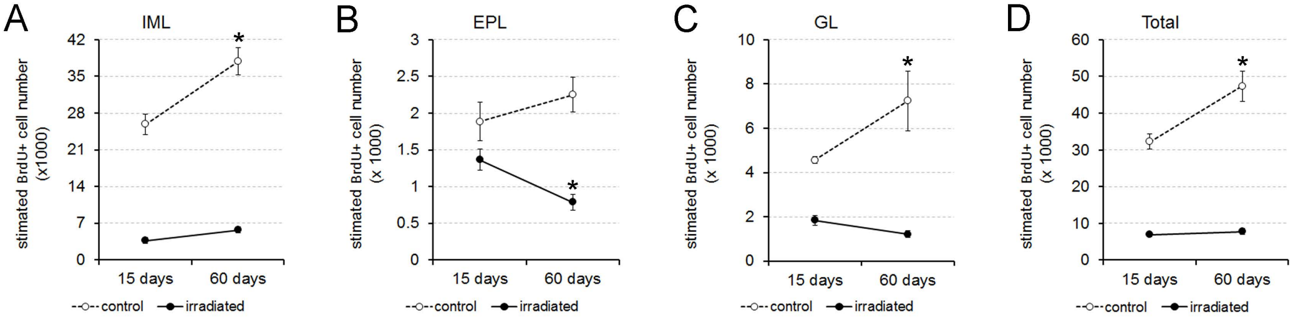
A
IML
B
EPL
C
GL
D
Total
stimated BrdU+ cell number (x1000)
stimated BrdU+ cell number (x 1000)
15 days
60 days
control
irradiated
*

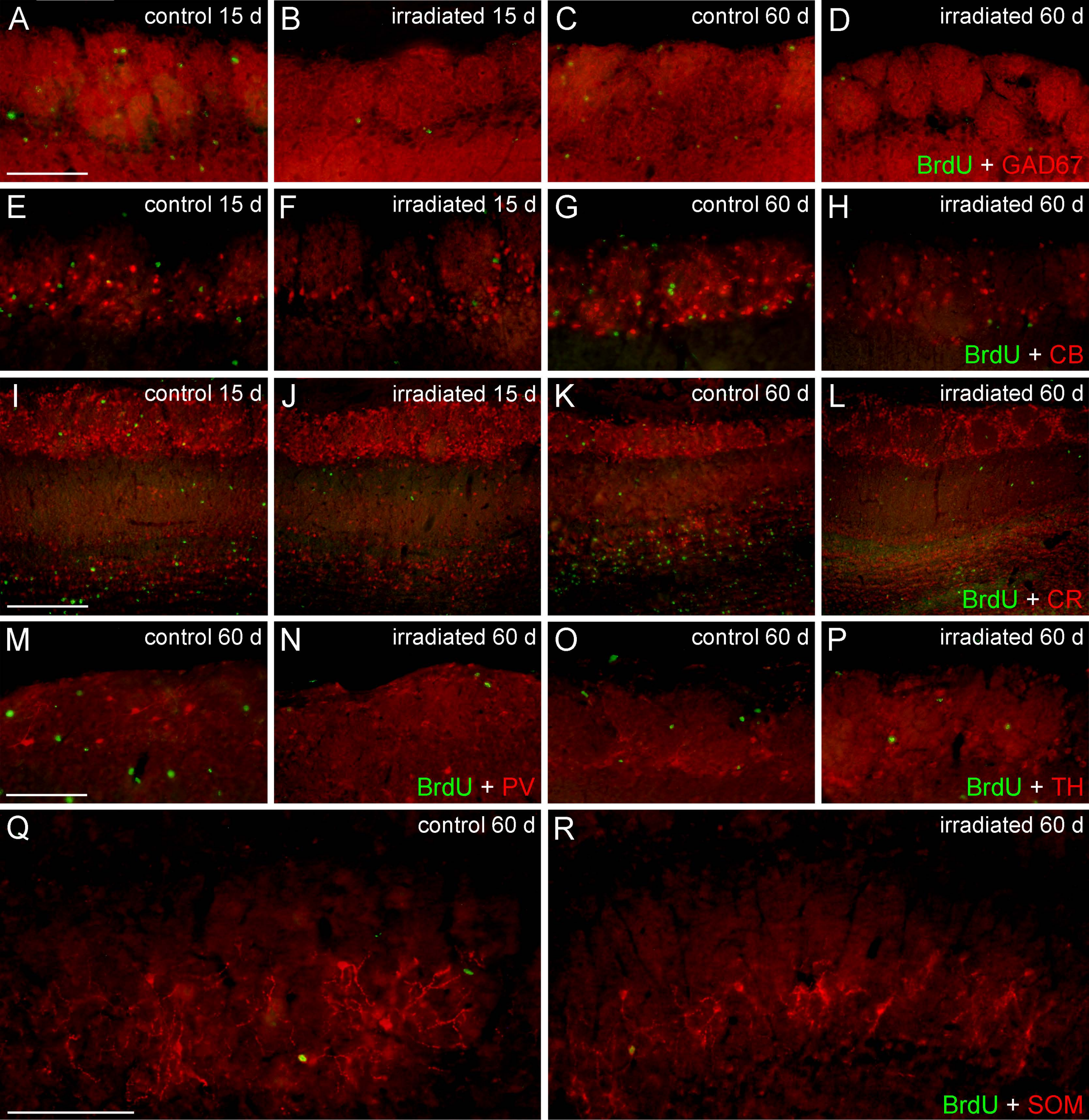
A control 15 d
B irradiated 15 d
C control 60 d
D irradiated 60 d
BrdU + GAD67
E control 15 d
F irradiated 15 d
G control 60 d
H irradiated 60 d
BrdU + CB
I control 15 d
J irradiated 15 d
K control 60 d
L irradiated 60 d
BrdU + CR
M control 60 d
N irradiated 60 d
BrdU + PV
O control 60 d
P irradiated 60 d
BrdU + TH
Q control 60 d
R irradiated 60 d
BrdU + SOM

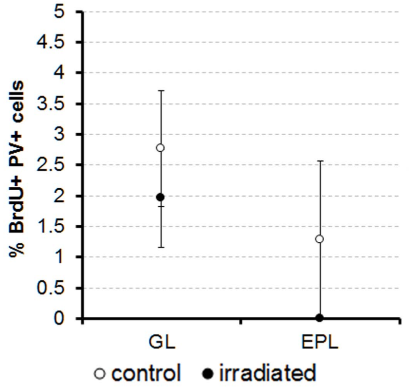

% BrdU+ PV+ cells
5
4.5
4
3.5
3
2.5
2
1.5
1
0.5
0
GL
EPL
control
irradiated
